# Ultrasound-Assisted Soaking Facilitates Purine Dissolution from Soybean Powder: Development and Preliminary Application of Low-Purine Soybean Powder

**DOI:** 10.3390/foods15101827

**Published:** 2026-05-21

**Authors:** Hongfeng Yu, Yuting Zheng, Lulu Yang, Yong Zhao, Xinxin Ma, Li Li, Haiquan Liu

**Affiliations:** 1 College of Food Science and Technology, Shanghai Ocean University, Shanghai 201306, China; yhf112186@163.com (H.Y.); zyt03995@163.com (Y.Z.); llyangi@163.com (L.Y.); yzhao@shou.edu.cn (Y.Z.); 2 Shanghai Engineering Research Center of Aquatic-Product Processing & Preservation, Shanghai 201306, China; 3 Laboratory of Quality & Safety Risk Assessment for Aquatic Product on Storage and Preservation (Shanghai), Ministry of Agriculture and Rural Affairs, Shanghai 201306, China; 4 Shanghai Tramy Green Food (Group) Co., Ltd., Shanghai 201300, China; qingmeimxx@163.com; 5 Engineering Research Center of Food Thermal-Processing Technology, Shanghai Ocean University, Shanghai 201306, China

**Keywords:** soybean, purine reduction, ultrasound-assisted soaking, low purine soy products

## Abstract

People suffering from gout and hyperuricemia have limited consumption of soy products because of their high purine content, even though soybean is a nutrient-rich crop. This study developed a combined purine reduction process: ultrasonic-assisted soaking to promote purine dissolution and isoelectric point precipitation to separate purines with minimal protein loss. A high-performance liquid chromatography (HPLC) method for rapid purine determination was first established (R^2^ > 0.9999, RSD < 0.23%), thereby providing technical support for process optimization. Using soybean powder as the raw material, optimal ultrasonic conditions (58 °C, 250 W, 58 min) were identified, achieving a purine removal rate of 61.15% with a protein recovery of 94.23%. Scanning electron microscopy (SEM) and Fourier-transform infrared (FTIR) spectroscopy analyses revealed that ultrasonic treatment altered the microstructure of the soybean powder, thereby facilitating purine dissolution. Low-purine soymilk prepared from the resulting soybean powder exhibited a unique flavor, with enhanced electronic nose response signals of its flavor compounds. This process effectively reduces purine content while preserving soy protein and flavor, offering a feasible technical solution for the development and industrial application of low-purine soy products. However, challenges remain in process scale-up and in optimizing the balance between purine removal and nutrient retention.

## 1. Introduction

Soybean serves as a nutrient-dense crop and an indispensable source of high-quality plant protein, with a crude protein content of approximately 40% [1] and a complete spectrum of human-essential amino acids [2,3]. This makes soy products an important substitute for meat, eggs, and dairy products, particularly suitable for vegetarians and individuals with specific dietary needs. Beyond basic nutrients, soybeans are rich in unsaturated fatty acids and various bioactive compounds such as soy isoflavones. offering significant benefits for cardiovascular and gastrointestinal health, as well as regulating physiological functions [4,5,6].

Despite the superior nutritional profile and health benefits of soybeans and soybean products, they contain relatively high levels of purines. The purine content in raw soybeans is approximately 150–200 mg/100 g [7], making them unsuitable for individuals with gout or hyperuricemia. The predominant purines in soybeans are adenine and guanine, which are metabolized into uric acid under the catalysis of xanthine oxidase in vivo. Uric acid in humans is primarily excreted through the kidneys (70%) and intestines (30%). Under normal conditions, the human body maintains a balance between uric acid absorption and excretion [8]. Nevertheless, abnormal uric acid metabolism or impaired function of metabolic organs in hyperuricemia and gout patients disrupts this homeostasis, leading to overproduction or insufficient excretion of uric acid and subsequent elevation of serum uric acid [9,10]. When the human body maintains a persistently supersaturated uric acid level (>6.8 mg/dL) [11], around 10% of circulating uric acid crystallizes into insoluble needle-shaped monosodium urate. These crystals eventually deposit in joints or soft tissues, triggering acute inflammatory arthritis, namely gout flares, which may be accompanied by complications such as chronic joint damage and kidney stones. Therefore, traditional dietary guidelines generally recommend that patients with hyperuricemia and gout limit or avoid the intake of high purine foods. Although most soybean products are moderate to low purine foods, the human dietary system is complex, and gout patients also need to consume a variety of foods. Reducing the purine levels in soy products allows gout patients to safely consume soy foods without additional dietary restrictions on other daily ingredients.

Given the urgent demand to reduce purine content while maximally retaining intrinsic nutrients, the exploration of efficient purine-reduction strategies has become a research hotspot in food science and nutrition for enabling safe soybean consumption among populations with hyperuricemia and gout. Current methods for lowering purine content in foods primarily fall into three categories: First, physical processing methods such as steaming, boiling, and soaking [12]. Purine removal through water solubilization is applied in both soy products and meat. For instance, Zheng et al. demonstrated that preliminary processing steps like soaking can reduce purine content in soy products by approximately 50%, effectively lowering purine levels [7]. Similarly, Monika Sabolová et al. demonstrated that boiling for 15 min significantly reduces purine levels in edible insects [13]. However, these conventional approaches inevitably cause massive losses of water-soluble nutrients, especially soluble proteins. Therefore, minimizing protein loss while reducing purine content is crucial for low-purine soy product processing. Second is bioconversion, encompassing microbial fermentation [14] and the use of purine-degrading enzymes. The latter has demonstrated potential in degrading purines in yeast, beef, and beer [15], but these methods require strict process control and significantly impact food flavor, limiting their application in soy product manufacturing. The third category covers novel physicochemical separation technologies such as salting-out and activated carbon adsorption [16,17], which are hindered by high costs and poor production scalability. Therefore, current research should focus on developing efficient, low-cost integrated processes. While evaluating the removal efficiency of combined technologies for purines in foods like soybeans, it is essential to simultaneously assess changes in nutrient retention rates, protein functional properties, and sensory quality. This will provide a theoretical basis and practical solutions for the development of functional foods with both low purine content and high nutritional value.

This study innovatively proposes a method combining ultrasonic soaking and isoelectric point adjustment to reduce purine content in soybean powder. Ultrasound cavitation and soaking thermal effects were synergistically applied to accelerate purine dissolution. This is followed by isoelectric point adjustment of soy protein to retain soluble proteins while separating and removing the purine solution. Combined with high-performance liquid chromatography detection technology, this method enables rapid assessment of purine content in samples, providing a purine detection approach for the development of low-purine soybean powder. The resulting low-purine soybean powder can be utilized in the production and processing of various soy products. Compared with traditional physical processing, the proposed technology markedly mitigates protein loss during purine removal, and provides a feasible and promising approach for developing high-nutrition low-purine functional soy foods.

## 2. Materials and Methods

### 2.1. Materials and Reagents

The soybean raw material used in this study was supplied by Shanghai Tramy Green Food (Group) Co., Ltd. (Shanghai, China). Four purine standards, including guanine, adenine, xanthine and hypoxanthine, were of chromatographic grade and purchased from Shanghai Macklin Biochemical Technology Co., Ltd. (Shanghai, China). Methanol of HPLC grade was purchased from TanMo Reference Materials Company Ltd. (Changzhou, Jiangsu, China). Formic acid was obtained from Xingfei Chemical Co., Ltd. (Zibo, China). Trifluoroacetic acid was purchased from Thermo Fisher Scientific (Waltham, MA, USA). NaOH, KH_2_PO_4_, and H_3_PO_4_ were all purchased from Guangzhou Chemical Reagent Factory (Guangzhou, China). The ultrapure water for all experiments was prepared by the MING-CHE 24 Ultraviolet Ultrapure Water System (Millipore, Burlington, MA, USA).

### 2.2. Ultrasonic Soaking Treatment of Soybean Powder

Soybean material was pulverized using a Midea LZ121 Multi-Function Cell-Breaking Machine (Foshan, China) and passed through a 40-mesh sieve. Then, 1.000 g of the powder was mixed with 30 mL of ultrapure water in a 50 mL centrifuge tube, shaken, and soaked at room temperature for 12 h.

After pretreatment, the soybean powder solution was ultrasonicated (ZX-300DE, Shanghai, China) at ultrasonic temperature (20–80 °C), ultrasonic power (0–300 W), ultrasonic time (20–70 min), which covered the ultrasonic intensity range adopted in the single-factor experiments. Each treatment was performed in triplicate.

After ultrasonication, the solution was centrifuged for 10 min at 4 °C and 8804× *g* using the Zhongke Meiling CT-G185R high-speed refrigerated centrifuge (Hefei, China). Two fractions were obtained: an upper liquid layer and a lower solid pellet.

Lower solid pellet: Collected and set aside.

Upper liquid layer: The upper layer was adjusted to pH 4.5 with 1 mol/L HCl according to Ma et al. [18] and centrifuged again (4 °C, 8804× *g*, 10 min). After centrifugation, 2 mL of the supernatant was pipetted and stored at 0–4 °C for HPLC analysis of purines within 12 h. while the precipitate was washed with ultrapure water and combined with the previously collected pellet.

### 2.3. Single-Factor and Response Surface Experiments

Based on ultrasonic soaking treatment, single-factor experiments were performed to explore the effects of ultrasonic temperature, power and treatment time on the purine removal rate of soybeans, aiming to screen the optimal parameter ranges for subsequent response surface optimization. The gradient settings of each single factor are presented below:

Temperature: 30, 40, 50, 60, 70, 80 °C.

Power: 0, 60, 120, 180, 240, 300 W.

Time: 20, 30, 40, 50, 60, 70 min.

Based on the single-factor experiment, the following response surface experiment was designed (Table 1), with the response value being the purine removal rate. The removal rate is calculated using Equation (1):

(1) $$Purine\;Removal\;Rate\left(\%\right)=\frac{C-C_{1}}{C}\times 100$$

*C* represents the purine content (mg/100 g) of the control soybean powder, *C*_1_ represents the purine content (mg/100 g) of the soybean powder after treatment, and 100 is the conversion factor.

**Table 1:** Response Surface Design.

Factor	Level		
	−1	0	1
A: Temperature (°C)	50	60	70
B: Power (W)	180	240	300
C: Time (min)	50	60	70

### 2.4. Establishment of a High-Performance Liquid Chromatography Method for Purine Detection

In this study, an Agilent ZORBAX Eclipse Plus C18 column (250 mm × 4.6 mm, 5 μm) was used to determine the purine contents in samples via an Agilent 1260 Infinity II HPLC system (Agilent Technologies Inc., Santa Clara, CA, USA). The mobile phase consisted of 10 mmol/L KH_2_PO_4_ solution (pH = 3.84) and methanol at a volume ratio of 98:2 (*v*/*v*). This pH and eluent ratio were selected to achieve good chromatographic separation of the four purine isomers under isocratic elution conditions. The flow rate was set at 1 mL/min, column temperature at 30 °C, injection volume at 10 μL, detection wavelength at 254 nm, and the total elution time was 25 min. Four purine standards, including guanine, adenine, xanthine and hypoxanthine (Macklin, Shanghai, China), were accurately prepared into a mixed stock solution of 100 μg/mL, which was stored at 0–4 °C for no more than 6 months. Before use, the stock solution was serially diluted to obtain working solutions at concentrations of 0.1, 0.5, 1, 5, 10, 20 and 50 μg/mL. All working solutions were freshly prepared, and the standard curve was established simultaneously with sample detection [19].

### 2.5. Determination of Purine Content in Samples

Soybean powder: Following the method of Lu et al. [20] with appropriate optimization, 0.5 g of the sample was accurately weighed into a 50 mL centrifuge tube, add 7.5 mL of sample extraction solution (trifluoroacetic acid: formic acid = 2:1, *v*/*v*), vortex to mix, and digest in a 95 °C water bath for 60 min to ensure sufficient purine extraction efficiency [21]. Immediately after digestion, the centrifuge tube was removed and cooled to room temperature in an ice bath. Transfer 1 mL of the supernatant to a 15 mL centrifuge tube and evaporate to near dryness using a DJ100-24D nitrogen evaporator (Hangzhou Deju Instrument Equipment Co., Ltd., Hangzhou, China). Resuspend in 3.5 mL of mobile phase, adjust the pH to 6–7, and dilute to a final volume of 4 mL. The mixture was centrifuged at 8804× *g* for 10 min. After centrifugation, transfer the supernatant through a 0.22 μm aqueous phase filter membrane into a sample vial for HPLC analysis. The purine content in soybean powder was calculated using Equation (2):

(2) $$\;X=\frac{k\times C\times V}{m}\times 10^{-1}$$

*X* represents the purine content in the sample (mg/100 g); *C* represents the purine concentration detected by HPLC (mg/L); *V* represents the final volume of the injection solution (mL); *m* represents the sample mass (g); *k* represents the sample dilution factor; 10^−1^ represents the unit conversion factor.

Soy milk and supernatant: Pipette 2 mL of sample into a 50 mL centrifuge tube, add 7.5 mL of extraction solution, digest in a 95 °C water bath for 30 min, and then follow the same steps as for soybean powder. The purine concentration is calculated using the following Equation.

*Y* = *k* × *C* × *V* (3)

*Y* represents the purine concentration in the sample (mg/L); *C* represents the purine concentration detected by HPLC (mg/L); *V* represents the final volume of the injection solution (mL); *k* represents the sample dilution factor.

### 2.6. Determination of Protein Content in Soybean Powder

The protein content in soybean powder was determined via the Kjeldahl method [22]. Approximately 0.2 g of the sample was accurately weighed and placed into a Kjeldahl digestion tube, followed by the addition of 6 g of potassium sulfate (K_2_SO_4_), 0.4 g of copper sulfate (CuSO_4_), and 20 mL of sulfuric acid (H_2_SO_4_). The mixture was digested in a Kjeldahl digestion furnace until the solution turned clear and exhibited a blue-green color. Subsequently, the digested sample was then analyzed using an automatic Kjeldahl nitrogen analyzer (KDN-16K, Shanghai Xianjian Instrument Co., Ltd., Shanghai, China). A nitrogen-to-protein conversion factor of 6.25 was applied for soybean. The instrument parameters were adjusted accordingly, and the final protein content was calculated.

### 2.7. Scanning Electron Microscopy (SEM)

Three groups of samples were imaged: the single soaking group (soaking at room temperature), the heated soaking group (58 °C, 0 W, 58 min), and the ultrasonic heated soaking group (58 °C, 250 W, 58 min, this process was conducted under the optimal conditions identified in response surface experiments, see Section 3.3 for details). After processing, each group of samples was freeze-dried using a Ye Tuo YTLG-10A freeze dryer (Shanghai, China). Following freeze-drying, an appropriate amount of each sample group was evenly spread onto a conductive adhesive. Subsequently, each sample was gold-sputtered for 60 s to enhance electrical conductivity for microscopic observation [23]. The gold-plated samples were then observed under a Hitachi SU5000 thermal field emission scanning electron microscope (Hitachi, Tokyo, Japan) to examine their microstructure. The acceleration voltage was set at 10 kV, with a magnification of 90×.

### 2.8. Fourier Transform Infrared (FTIR) Spectroscopy Analysis

Four groups of dried samples were tested using a Thermo Nicolet iS 10 Fourier transform infrared spectrometer (Waltham, MA, USA), namely the control group (dry soybean powder), single soaking group (soaking at room temperature), heated soaking group (58 °C, 0 W, 58 min), and ultrasonic heated soaking group (58 °C, 250 W, 58 min), The spectral range of 4000–400 cm^−1^ and 32 scans were set to obtain stable and high-quality infrared spectra for protein secondary structure analysis. The amide I band at 1600–1700 cm^−1^ was further deconvoluted to quantify the relative proportions of protein secondary structures [24]. After deconvolution using PeakFit v4.12 software, the second derivative was calculated to determine the proportions of α-helix, β-sheet, random coil, and β-turn structures of the protein [25].

### 2.9. Preparation of Soymilk

Three groups of dried samples: single soaking group (soaking at room temperature), heated soaking group (58 °C, 0 W, 58 min), and ultrasonic heated soaking group (58 °C, 250 W, 58 min), were used as raw materials to prepare soymilk. The weighed soybean powder was added to distilled water at a mass ratio of soybean powder to water of 1:10. Referring to the method described by Mu et al. [26], after grinding, the soymilk was boiled at 95 °C for 15 min [27]. After cooling, it was stored in a refrigerator at 4 °C for subsequent determinations.

### 2.10. Electronic Nose Analysis

Following the methodology described by Zong et al. [28], a Supernose electronic nose system (Baosheng, Shanghai, China) was employed to analyze the flavor characteristics of soymilk. Freshly prepared soymilk was transferred into 25 mL headspace vials and hermetically sealed to allow volatile flavor compounds to accumulate in the headspace. Before measurement, the probes were purged for 100 s and calibrated for 5 s, with an additional 5 s of equilibration to stabilize the sensor baseline. A carrier gas flow rate of 300 mL/min and a sampling duration of 60 s were adopted to ensure sufficient volatile capture and stable signal acquisition. All determinations were carried out in triplicate [29]. The electronic nose system was equipped with 18 sensors with the following specificities: S1: alkanes and smoke; S2: alcohols, aldehydes, and short-chain alkanes; S3: ozone; S4: sulfide, hydrogen sulfide; S5: nitrogen, ammonia; S6: Organic gases, Benzones, Aldehydes, Aromatic compounds, S7: Short-chain alkanes, Natural gas, Biogas; S8: Short chain alkanes; S9: Short chain alkanes; S10: Hydrogen;S11: Allyl sulfide; S12: Ketones, Alcohols; S13: Methane; S14: Combustible gases; S15: Volatile organic compounds, Abnormal odor; S16: Butane, Liquefied gas; S17: Methane, Natural gas; S18: Propane, Butane.

### 2.11. Statistical Analysis

Data analysis and graphing were performed using SPSS Statistics 26.0 and Origin Pro 2021, respectively. All data were presented as the mean ± standard deviation (SD). One-way analysis of variance (ANOVA) was adopted to evaluate significant differences among measured indicators, with the significance level set at *p* < 0.05.

## 3. Results and Discussion

### 3.1. Validation of High-Performance Liquid Chromatography Method

**Figure 1:**
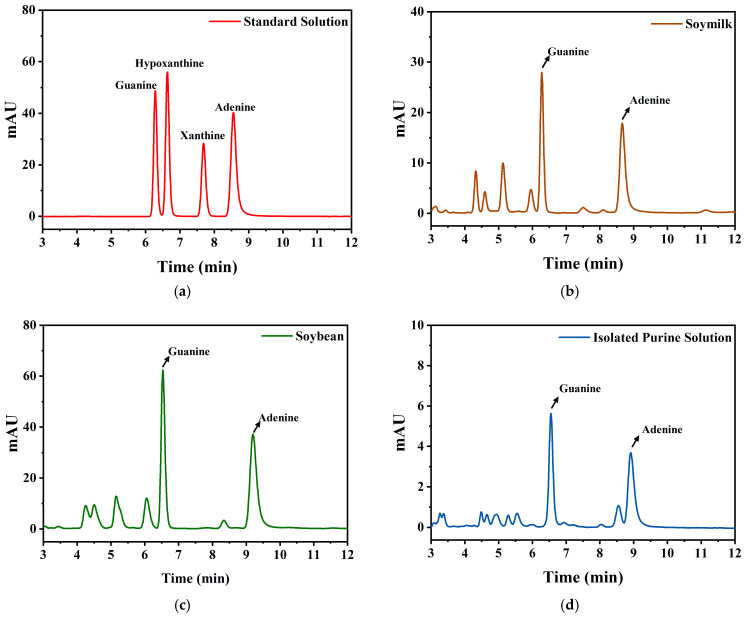
HPLC chromatograms of standard solutions and samples. (**a**) 10 ppm mixed purine standard solution, (**b**) Soy milk, (**c**) Soybean, (**d**) Isolated purine solution.

By optimizing chromatographic conditions, four purines (guanine, hypoxanthine, xanthine and adenine) were effectively separated in this study. The liquid chromatogram of the mixed standard solution is presented in Figure 1a, which exhibits symmetric peak shapes, stable retention times and satisfactory separation resolution for individual components. This lays a solid foundation for the accurate quantitative analysis and further development of low-purine soybean powder. Subsequently, standard curves of the four purines were established. All correlation coefficients (R^2^) of the linear regression equations were greater than 0.9999 (Table 2), with a linear range spanning 0.1–50 μg/mL. The relative standard deviation (RSD%) was less than 0.23%, confirming favorable linearity and precision [30,31], capable of meeting precise quantitative demands for purine in samples of varying concentrations. Furthermore, the developed method was applied to actual samples, including soymilk, soybeans and isolated purine solutions (Figure 1b–d). The purine components in real samples showed highly consistent retention times with standard solutions, and no obvious interfering peaks were observed. This provides a stable and reliable analytical approach for subsequent purine compound analysis.

### 3.2. Single-Factor Experiment on Ultrasonic Soaking Process

**Figure 2:**
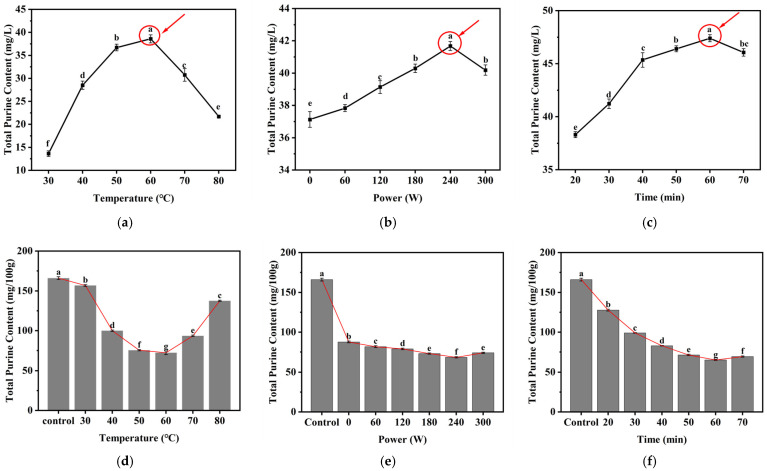
Effect of single-factor process parameters on purine removal efficiency. (**a**–**c**) show the variation patterns of purine concentration in the supernatant with respect to (**a**) ultrasonic temperature, (**b**) ultrasonic power, and (**c**) ultrasonic time. (**d**–**f**) show the variation patterns of purine concentration in soybean powder with respect to (**d**) ultrasonic temperature, (**e**) ultrasonic power, and (**f**) ultrasonic time. (Note: The red circle marks the point with the maximum purine dissolution in the single-factor experiment. Letters indicate statistical significance, *p* < 0.05).

The results of single-factor experiment are shown in Figure 2, each processing parameter presented a distinct influence on purine removal. In the purine supernatant (Figure 2a–c), the total purine content showed a continuous upward trend when the ultrasonic temperature increased from 30 to 60 °C, ultrasonic power elevated from 0 to 240 W, and sonication time prolonged from 20 to 60 min. The maximum purine content was obtained at 60 °C, 240 W, and 60 min, respectively. Further increases in temperature, power, or time led to a decline in supernatant purine content to different extents. On the contrast, the total purine content in soybean powder (Figure 2d–f) decreased with the increase in temperature, power, and time, reaching its lowest levels at 60 °C, 240 W, and 60 min.

Peng et al. [32] demonstrated that soybean 7S globulin was fully denatured at temperatures above 70 °C. Consequently, excessively high temperatures may cause denatured aggregation of soy proteins, thereby hindering the release of purines from soybean powder. This phenomenon well explains the decreased purine level in the supernatant and the rebound of residual purines in soybean powder when the temperature exceeded 60 °C. Excessively high ultrasonic power or prolonged sonication time may cause a slight rise in purine content in soybean powder. Excessive ultrasonic power can irreversibly destroy the microscopic structure of soybean powder, accelerate protein aggregation, and ultimately reduce the extraction efficiency of purines [33]. During prolonged ultrasonic treatment, small molecules like purine dissolved into the supernatant may be adsorbed or complexed by macromolecules released from the soybean through hydrophobic interactions, hydrogen bonding, or electrostatic complexation. Such interactions reduce the purine concentration in the supernatant and simultaneously result in a slight accumulation of residual purines in soybean powder [16]. The results indicate that under the optimal ultrasonication conditions of 60 °C, 240 W and 60 min, the transfer efficiency of purine from soybean powder to the supernatant is maximized, achieving the most significant purine removal effect.

### 3.3. Response Surface Optimization for Ultrasonic Soaking

Using Design-Expert 13 software, a multiple regression fit was performed on the experimental results in Table 3, yielding the quadratic polynomial regression Equation (4):

Y = 60.88 − 2.52A + 0.76B − 0.21C + 0.67AB + 0.85AC − 0.43BC − 5.53A^2^ − 3.01B^2^ − 2.85C^2^ (4)

**Figure 3:**
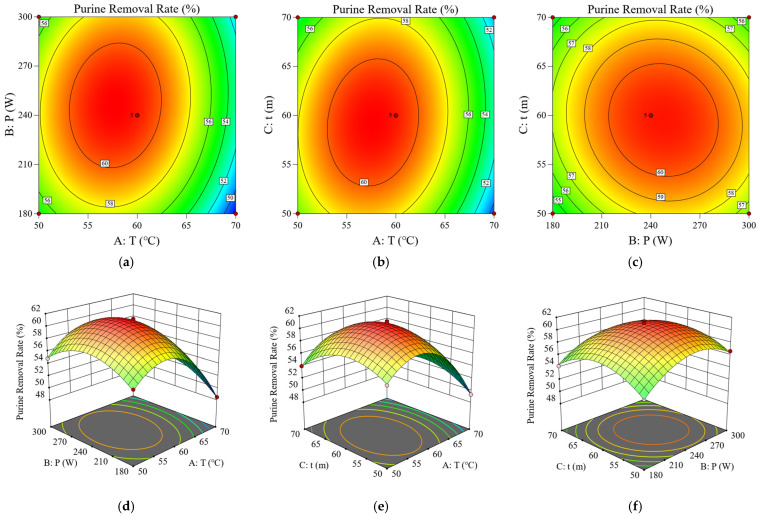
Response surface and contour plots for optimizing the purine reduction process. The response value represents the purine removal rate in soybean powder: (**a**,**d**) ultrasonic temperature versus ultrasonic power; (**b**,**e**) ultrasonic temperature versus ultrasonic time; (**c**,**f**) ultrasonic power versus ultrasonic time.

The established response surface model exhibits high significance (*p* < 0.0001). The lack of Fit value was not significant (*p* = 0.6410 > 0.01), with a coefficient of determination R^2^ = 0.9972. These results demonstrated an excellent fitting degree of the model, which could accurately predict the process efficacy of ultrasonic-assisted purine removal from soybean powder [34,35]. Based on the coefficient analysis of the regression equation 4 and significance evaluation (Table 4), the influencing order of the three variables on the purine removal rate was determined as follows: ultrasonic temperature (A) > ultrasonic power (B) > ultrasonic time (C). This finding was further supported by the response surface plots (Figure 3). As shown in Figure 3a,d, there was a strong interaction between temperature and power, and temperature acted as the predominant factor. The purine removal rate reaches its peak near 60 °C and 240 W. Figure 3b,e indicated a notable interaction between temperature and time, where temperature played a dominant role while time exerted a relatively weak effect. The peak purine removal rate was achieved near 60 °C and 60 min. In Figure 3c,f the contour lines of power and time were nearly circular, indicating a mild interaction between them. Meanwhile, their individual effects on the purine removal rate were markedly lower than that of temperature [36].

With the regression model, the optimal treatment conditions for maximum purine removal were determined: ultrasonic temperature of 58.18 °C, ultrasonic power of 249.55 W, and ultrasonic time of 58.19 min. Under the above conditions, the theoretical predicted removal rate was 61.20%. Considering operational feasibility, the parameters were appropriately adjusted to 58 °C, 250 W, and 58 min for practical verification. Experimental verification under these conditions yielded an actual purine removal rate of 61.15%. This validated the model’s rationality and reliability [37], conferring practical significance. Subsequently, the experimental conditions derived from this model were applied in follow-up experiments.

### 3.4. Protein Retention and Structural Changes

#### 3.4.1. Protein Retention

As illustrated in Table 5, compared with the control group, after ultrasound treatment without pH adjustment, the protein content of the soybean powder solid obtained from the first centrifugation (protein content: 25.22 ± 1.29%) was significantly reduced, because a large proportion of soybean protein was dissolved in water and removed together with purines without being retained. After adjusting the pH of the solution to the isoelectric point of soybean protein and performing a second centrifugation, the soybean protein was retained, and its content recovered to 40.48 ± 0.73%, with a recovery rate as high as 94.23%.

#### 3.4.2. Microstructure of Soybean and Changes in Protein Secondary Structure

**Figure 4:**
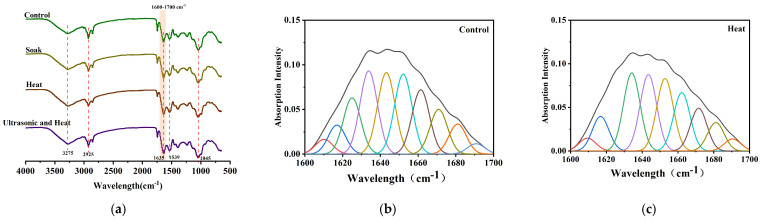

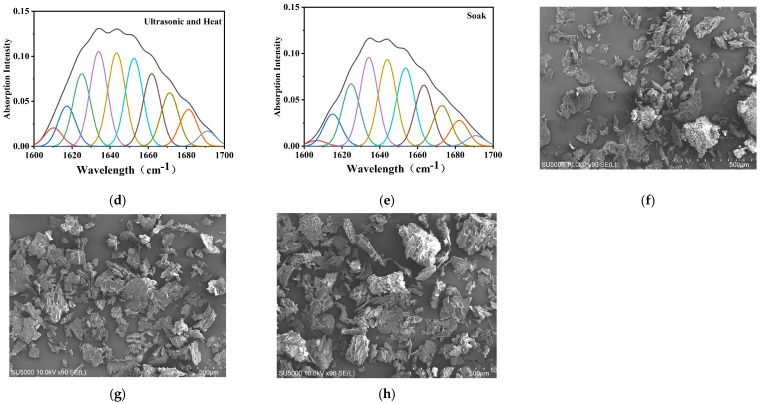
Secondary structure analysis and microstructure images under different treatment conditions. (**a**) FT−IR Spectra of soybean powder. (**b**–**e**) Second-derivative fitting plots of soybean powder at 1600–1700 cm^−1^. (**f**–**h**) Scanning electron microscope images of soybean powder from the soak group, heat group, ultrasonic and heat group.

Under different treatment conditions, the secondary structure and microstructure of soybean protein changed significantly, whereas complete protein denaturation did not occur [38]. These changes may be closely related to purine dissolution behavior. Based on Fourier transform infrared spectroscopy results (Figure 4a) and peak fitting analysis (Figure 4b–e, Table 6), compared to the control group, room-temperature soaking increased the relative contents of β-sheet and random coils, and slightly reduced α-helix and β-turn fractions when compared with the control group. Although water penetration-induced hydrogen bond rearrangement loosened the soybean powder structure [39] and facilitated the release of bound purines, diffusion efficiency may have been limited due to low temperature, resulting in lower release efficiency. Soaking at 60 °C further raised the β-sheet content, declined random coil and α-helix proportions, and slightly restored β-turns. The thermal effect disrupted hydrogen bonds, unfolding peptide chains to release encapsulated purines. However, excessive β-sheet formation may cause peptide chain aggregation into dense networks, potentially re-encapsulating purines or hindering their diffusion [40]. This explains the decrease in dissolution rate beyond 60 °C (Figure 2a,d). Ultrasound-assisted thermal soaking yielded the higher β-sheet proportion and lowest α-helix proportion. The cavitation effect and shear force of ultrasonication vigorously disrupted protein structure, promoting intermolecular hydrogen bond formation and inducing substantial β-sheet generation. Despite the potential risk of purine encapsulation caused by protein aggregation, the generated open aggregate structure greatly favored purine release [23].

Such structural differences were further verified by SEM images. As shown in Figure 4f–h, the three sample groups exhibited markedly different microstructures. The soybean powder particles in the room-temperature soaking group (Figure 4f) had relatively smooth and intact surfaces, with only a few minor cracks caused by water penetration. In contrast, the particles in the heating-only group (Figure 4g) displayed rough surfaces, obvious flaky protrusions, and signs of partial melting and adhesion, demonstrating heat-induced protein aggregation and structural collapse—a morphology unfavorable for the thorough release of internal substances. Notably, the ultrasound assisted heating group (Figure 4h) presented a fundamentally different, loose, porous structure. This structure significantly enhanced purine dissolution efficiency, thereby establishing the structural basis for the efficient purine reduction performance of this process.

### 3.5. Soy Milk Flavor Assessment

**Figure 5:**
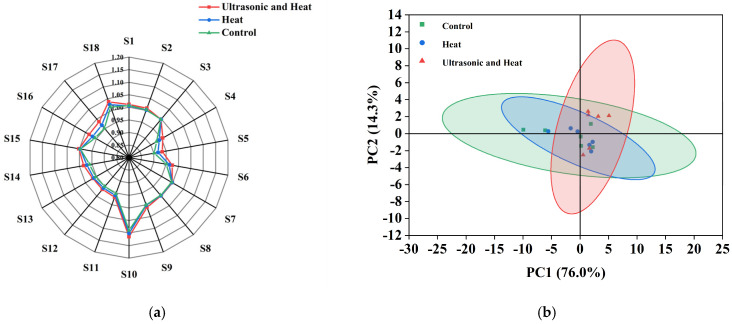
Flavor evaluation of low−purine soy milk. (**a**) Radar chart from the electronic nose. (**b**) PCA analysis diagram from the electronic nose.

This study employed electronic nose technology combined with principal component analysis (PCA) to systematically investigate the effects of three treatments (namely ultrasonic and heat treatment, heat treatment, and a control group) on the volatile flavor compounds of soy milk. The electronic nose radar chart (Figure 5a) intuitively presents the response differences of 18 sensors among different groups, and the PCA score plot (Figure 5b) further quantified the overall flavor discrimination of each treatment.

The radar chart demonstrated distinct sensor responses among the three groups. Overall, the ultrasonic-assisted heating group obtained higher response values than the heat and control groups. Specifically, sensor S2 is sensitive to alcohols and aldehydes, which are closely associated with soymilk quality and palatability [41]. The result suggested that ultrasonic treatment might contribute to the release of related flavor substances. Moreover, the ultrasonic-assisted heating group showed remarkably stronger signals on sensor S4 (sulfur compounds), S5 (nitrogen and ammonia substances), and S6 (aromatic compounds) compared with the other two groups. These signal differences suggested that ultrasonic-assisted heating could alter the overall flavor profile by regulating the release of sulfur-containing, nitrogenous and aromatic flavor-related substances, which are vital to the characteristic flavor of soymilk [42].

As indicated by the PCA results, the cumulative variance contribution rate of PC1 and PC2 reached 90.3%, indicating that the first two principal components could sufficiently reflect the main information of the original data. All samples were well separated in the PCA space, revealing that different processing methods led to obvious differences in the overall flavor profile of soymilk [43]. The ultrasonic-assisted heating group showed clear separation from the control, implying a more noticeable change in overall flavor characteristics. The single heating group also exhibited some separation from the control, suggesting that heating alone can induce flavor changes, albeit to a lesser extent than ultrasonic-assisted heating.

Overall, electronic nose combined with PCA analysis confirmed that ultrasonic-assisted heating altered the overall flavor profile of soymilk, presenting a more distinct flavor regulation effect than single heating treatment. The cavitation effect of ultrasound may disrupt macromolecular structures such as proteins and fats in soybean powder, promoting the release and conversion of flavor precursors [44]. This process may facilitate the generation and volatilization of flavor substances, and may also intensify undesirable flavor formation. This work provides a preliminary reference for the processing optimization and flavor regulation of low-purine soymilk. In future studies, gas chromatography-mass spectrometry (GC-MS) will be adopted to qualitatively and quantitatively characterize key volatile compounds, so as to further clarify the intrinsic flavor formation mechanism under ultrasonic-assisted heating.

## 4. Conclusions

This study systematically established and optimized an ultrasonic soaking strategy to reduce purine content in soybean powder, aiming to address the dietary limitations for susceptible populations caused by high purine levels in soybeans. Firstly, a high-performance liquid chromatography (HPLC) method was developed for purine quantification, which achieved effective separation of four purine monomers. This method demonstrated good linearity and high precision, providing reliable analytical support for subsequent experiments. Single-factor experiments clarified the influence patterns of ultrasonic temperature, power, and time on purine removal efficiency. Response surface methodology optimized the conditions to: ultrasonic temperature 58 °C, power 250 W, duration 58 min, achieving a purine removal rate of 61.15% with a protein recovery of 94.88% under these parameters.

Microstructural and protein secondary structure analysis confirmed that ultrasonic treatment induced a loose and porous microstructure in soybean powder and regulated protein conformational transformation, which collectively facilitated purine release and dissolution. These findings clarified the underlying mechanism of ultrasonic soaking in purine reduction. Furthermore, ultrasonic soaking was found to affect the flavor profile of soymilk by regulating the release of characteristic volatile compounds.

Our study innovatively integrates ultrasound-assisted soaking with isoelectric precipitation to efficiently remove purines while preserving high protein retention in soybean powder. Microstructural and flavor analyses reveal the underlying mechanism, and soymilk preparation verifies its practical application potential. However, protein characterization in this study is limited to secondary structure analysis only, and the optimization strategy and depth of flavor analysis also present certain limitations. Therefore, further research is required to conduct more comprehensive analyses of protein structure, functional properties, and flavor mechanisms. Future work will comprehensively evaluate product storage stability, sensory performance, and functional properties to facilitate industrial translation.

## Figures and Tables

**Table 2:** Standard Curve for Four Purines.

Name	Standard Curve	R^2^	Linearity Rang (μg/mL)	RSD%
Guanine	Y = 36.0413x − 1.9415	0.99999	0.1~50	0.16
Hypoxanthine	Y = 43.3422x − 2.0862	0.99998	0.1~50	0.19
Xanthine	Y = 25.2017x − 0.7834	0.99999	0.1~50	0.23
Adenine	Y = 49.4763x − 2.4391	0.99999	0.1~50	0.23

**Table 3:** Response Surface Experimental.

Number	Temperature (°C)	Power (W)	Time (min)	Purine Removal Rate (%)
1	50	180	60	54.96
2	70	180	60	48.46
3	50	300	60	54.88
4	70	300	60	51.07
5	50	240	50	55.91
6	70	240	50	49.29
7	50	240	70	54.03
8	70	240	70	50.79
9	60	180	50	54.02
10	60	300	50	56.65
11	60	180	70	54.25
12	60	300	70	55.15
13	60	240	60	60.81
14	60	240	60	61.09
15	60	240	60	60.27
16	60	240	60	61.17
17	60	240	60	61.07

**Table 4:** Regression Model Analysis of Variance.

Source	Sum of Squares	df	Mean Square	F-Value	*p*-Value	
Model	282.71	9	31.41	278.12	<0.0001	significant
A	50.85	1	50.85	450.26	<0.0001	
B	4.59	1	4.59	40.64	0.0004	
C	0.3403	1	0.3403	3.01	0.1262	
AB	1.81	1	1.81	16.02	0.0052	
AC	2.86	1	2.86	25.29	0.0015	
BC	0.7482	1	0.7482	6.62	0.0368	
A^2^	128.58	1	128.58	1138.40	<0.0001	
B^2^	38.24	1	38.24	338.55	<0.0001	
C^2^	34.22	1	34.22	303.02	<0.0001	
Residual	0.7906	7	0.1129			
Lack of Fit	0.2493	3	0.0831	0.6142	0.6410	not significant
Pure Error	0.5413	4	0.1353			
Cor Total	283.50	16				

**Table 5:** Protein content of Soybean.

Treatment	Protein Content (%)
control	42.96 ± 0.98 ^a^
1	25.22 ± 1.29 ^c^
2	40.48 ± 0.73 ^b^

Note: Letters indicate statistical significance, *p* < 0.05.

**Table 6:** Secondary Structure Analysis of Soybean Protein.

Treatment	β-Sheet (%)	Random Coil (%)	α-Helix (%)	β-Turn (%)
control	48.67 ± 0.05 ^c^	18.53 ± 0.43 ^b^	18.18 ± 0.06 ^a^	14.62 ± 0.15 ^a^
soak	49.06 ± 0.18 ^b^	19.54 ± 0.16 ^a^	17.65 ± 0.24 ^b^	13.75 ± 0.20 ^c^
heat	50.43 ± 0.27 ^a^	18.27 ± 0.24 ^b^	17.33 ± 0.05 ^c^	13.97 ± 0.05 ^bc^
Ultrasonic and soak	50.37 ± 0.17 ^a^	18.22 ± 0.15 ^b^	17.20 ± 0.13 ^c^	14.21 ± 0.17 ^b^

Note: Letters indicate statistical significance, *p* < 0.05.

## Data Availability

The original contributions presented in this study are included in the article. Further inquiries can be directed to the corresponding authors.

## References

[1] Kumar V., Rani A., Hussain L., Yadav M., Jha P., Petwal V., Dwivedi J. (2017). Changes in physico-chemical properties of native and toasted defatted soy flour on submission to electron beam radiation. Food Bioprod. Process..

[2] Shi D., Hang J., Neufeld J., Zhao S., House J.D. (2023). House, Effects of genotype, environment and their interaction on protein and amino acid contents in soybeans. Plant Sci..

[3] Handa C.L., Zhang Y., Kumari S., Xu J., Ida E.I., Chang S.K.C. (2020). Comparative Study of Angiotensin I-Converting Enzyme (ACE) Inhibition of Soy Foods as Affected by Processing Methods and Protein Isolation. Processes.

[4] Guan T., Liu X., Zhang L., Ren C., Feng Y., Yang Z., Xiao L. (2025). Soybean-Derived Bioactive Components in Prevention and Intervention of Lung Cancer. Mol. Nutr. Food Res..

[5] Belobrajdic D.P., James-Martin G., Jones D., Tran C.D. (2023). Soy and Gastrointestinal Health: A Review. Nutrients.

[6] Costa J.E.G., Azevedo P.Z., Matos J.d.S., Wischral D., Rigolon T.C.B., Stringheta P.C., Martins E., Campelo P.H. (2025). Strategies for Improving the Techno-Functional and Sensory Properties of Bean Protein. Processes.

[7] Zheng Y., Ma X., Li L., Yang L., Yu H., Zhao Y., Liu H. (2025). Purine content of different soybean products and dynamic transfer in food processing techniques. Food Chem. X.

[8] Han Q.-Q., Ren Q.-D., Guo X., Farag M.A., Zhang Y.-H., Zhang M.-Q., Chen Y.-Y., Sun S.-T., Sun J.-Y., Li N.-Y. (2025). Punicalagin attenuates hyperuricemia via restoring hyperuricemia-induced renal and intestinal dysfunctions. J. Adv. Res..

[9] Zhou D.-D., Zhang Q., Zhang H., Wang Y.-Z., Yang F.-Q., Wang S.-P., Wang Y.-T. (2019). Cupric ion functionalized polydopamine coated magnetic microspheres as solid-phase adsorbent for the extraction of purines in plasma. J. Chromatogr. B.

[10] Zou H., Xiang M., Ye X., Xiong Y., Xie B., Shao J. (2015). Reduction of urinary uric acid excretion in patients with proteinuria. J. Chromatogr. B.

[11] McCormick N., Yokose C., Challener G.J., Joshi A.D., Tanikella S., Choi H.K. (2024). Serum Urate and Recurrent Gout. JAMA.

[12] Li T., Ren L., Wang D., Song M., Li Q., Li J. (2020). Effect of allicin and its mechanism of action in purine removal in turbot. J. Food Sci..

[13] Sabolová M., Kulma M., Petříčková D., Kletečková K., Kouřimská L. (2023). Changes in purine and uric acid content in edible insects during culinary processing. Food Chem..

[14] Chu C., Zhou D., Men Y., Wang J., Zhang S., Fan H., He Y., Zhao X., Liu H. (2025). Targeted and untargeted metabolomics reveal dynamic purine metabolism in soy whey fermented by yeast. Food Chem..

[15] Wang C., Zhang R., Sun Y., Wen Y., Liu X., Xing X. (2023). Combinatorial co-expression of xanthine dehydrogenase and chaperone XdhC from Acinetobacter baumannii and *Rhodobacter capsulatus* and their applications in decreasing purine content in food. Food Sci. Hum. Wellness.

[16] Almeida C., Neves M.C., Freire M.G. (2021). Towards the Use of Adsorption Methods for the Removal of Purines from Beer. Molecules.

[17] Bercha S., Bhasker-Ranganath S., Zheng X., Beranová K., Vorokhta M., Acres R.G., Skála T., Matolín V., Prince K.C., Xu Y. (2020). Adsorption structure of adenine on cerium oxide. Appl. Surf. Sci..

[18] Ma H., Li J., Guan Y., Song Z., Chen H., Sun S. (2025). Structural and functional properties of soy protein isolates from different cultivars. Int. J. Biol. Macromol..

[19] Feng X., Ma H., Zou L., Wang Y., Zhang Y., Wang Y., Chen J., Pan H., Rong S. (2023). Determination of purines in prepackaged food using optimum acid hydrolysis followed by high performance liquid chromatography. Food Chem..

[20] Xiao L., Sha W., Tao C., Hou C., Xiao G., Ren J. (2022). Effect on purine releasement of *Lentinus edodes* by different food processing techniques. Food Chem. X.

[21] Wu M., Zhang W., Shen X., Wang W. (2021). Simultaneous Determination of Purines and Uric Acid in Chinese Chicken Broth Using TFA/FA Hydrolysis Coupled with HPLC-VWD. Foods.

[22] Amponsah A., Nayak B. (2016). Effects of Microwave and Ultrasound Assisted Extraction on the Recovery of Soy Proteins for Soy Allergen Detection. J. Food Sci..

[23] Zhang L., Hu Y., Wang X., Fakayode O.A., Ma H., Zhou C., Xia A., Li Q. (2021). Improving soaking efficiency of soybeans through sweeping frequency ultrasound assisted by parameters optimization. Ultrason. Sonochem..

[24] Tan L., Hong P., Yang P., Zhou C., Xiao D., Zhong T. (2019). Correlation Between the Water Solubility and Secondary Structure of Tilapia-Soybean Protein Co-Precipitates. Molecules.

[25] Wang Z., Zou P., Mi Q., Xu J. (2026). Effects of ultrasound-assisted non-covalently bound dietary antioxidants on the structure, flavor and digest characteristics of soymilk. Food Chem..

[26] Mu Q., Su H., Zhou Q., Xiao S., Zhu L., Xu X., Pan S., Hu H. (2022). Effect of ultrasound on functional properties, flavor characteristics, and storage stability of soybean milk. Food Chem..

[27] Shi X., Li J., Wang S., Zhang L., Qiu L., Han T., Wang Q., Chang S.K.-C., Guo S. (2015). Flavor characteristic analysis of soymilk prepared by different soybean cultivars and establishment of evaluation method of soybean cultivars suitable for soymilk processing. Food Chem..

[28] Zong L., Qu H., Wang W., Chen D., Wa Y., Huang Y., Gu R. (2025). Effect of key flavor compounds in fermented soymilk on sensory attributes: Integrating electronic sensory technology with GC–MS analysis. Food Chem. X.

[29] Liu Q., Lv Y., Zhou Y., Liu M., Feng H., Shen C., Wang H., Cao X., Kan J. (2025). Elucidation of Flavor Profile Dynamics in Tea-Flavor Baijiu During Long-Term Storage Using Sensory Evaluation, Electronic Nose. HS-GC-IMS, and HS-SPME-GC-MS. Processes.

[30] Krata A.A., Domagała J., Głowacki R. (2024). Hydrophilic interaction liquid chromatography based method for simultaneous determination of purines and their derivatives in food spices. Food Chem..

[31] Giuliani P., Zuccarini M., Buccella S., Rossini M., D’aLimonte I., Ciccarelli R., Marzo M., Marzo A., Di Iorio P., Caciagli F. (2016). Development of a new HPLC method using fluorescence detection without derivatization for determining purine nucleoside phosphorylase activity in human plasma. J. Chromatogr. B.

[32] Peng Y., Kyriakopoulou K., Rahmani A., Venema P., van der Goot A.J. (2021). Isochoric moisture heating as a tool to control the functionality of soy protein. LWT.

[33] Ma X., Hou F., Zhao H., Wang D., Chen W., Miao S., Liu D. (2020). Conjugation of soy protein isolate (SPI) with pectin by ultrasound treatment. Food Hydrocoll..

[34] Huang P.-H., Chiu C.-S., Chan Y.-J., Chen S.-J., Lu W.-C., Li P.-H. (2023). Response Surface Analysis and Process Optimisation of adzuki bean (*Vigna angularis*) food paste production. J. Agric. Food Res..

[35] Mhlongo J.T., Nuapia Y., Tlhaole B., Mahlangu O.T., Etale A. (2022). Optimization of Hemp Bast Microfiber Production Using Response Surface Modelling. Processes.

[36] Singh P., Bilyeu L., Krishnaswamy K. (2022). Spray drying process optimization: Drought resistant variety (W82) soymilk powder using response surface methodology (RSM). LWT.

[37] Zhu C., Zhou Y., Xie Q., Pan Y., Zhao Y., Liu H. (2025). Integration of E-tongue, GC-IMS and chemometrics for unraveling the disparities in volatile profiles of optimized jujube craft beers. J. Chromatogr. A.

[38] Vanga S.K., Wang J., Orsat V., Raghavan V. (2020). Effect of pulsed ultrasound, a green food processing technique, on the secondary structure and in-vitro digestibility of almond milk protein. Food Res. Int..

[39] Zhao X., Chen F., Xue W., Lee L. (2008). FTIR spectra studies on the secondary structures of 7S and 11S globulins from soybean proteins using AOT reverse micellar extraction. Food Hydrocoll..

[40] Wang J., Saxena R., Vanga S.K., Raghavan V. (2022). Effects of Microwaves, Ultrasonication, and Thermosonication on the Secondary Structure and Digestibility of Bovine Milk Protein. Foods.

[41] Zhang X., Tian W., Xie B., Sun Z. (2022). Insight into the Influence of Lactic Acid Bacteria Fermentation on the Variations in Flavor of Chickpea Milk. Foods.

[42] Anbarasan R., Dharini M., Jaganmohan R., Radhakrishnan M. (2023). Plasma Bubbling of Soymilk: Impact on Allergenicity, Antinutritional Factor, and E-Nose-Retrieved Sensory Characteristics. ACS Food Sci. Technol..

[43] Shinichiro H., Masayuki A., Takuya Y., Daisho Y., Atsushige F., Kana T., Miki M., Mito K., Yutaka K. (2024). Steam distillation process for flavor enhancement of milk coffee: Effects of condensation temperature on volatile compounds and flavor characteristics. J. Food Sci..

[44] Cheng L., Xiao H., Tu D., Zhao Y., Lin Y., Xiang Q., Tian Y. (2026). Study on the mechanism of flavor development in ultrasound-assisted microwave vacuum drying of shiitake mushrooms: Dosage effect of ultrasonic amplitude. Food Chem..

